# Changes of Retina Are Not Involved in the Genesis of Visual Hallucinations in Parkinson's Disease

**DOI:** 10.1155/2015/709191

**Published:** 2015-02-03

**Authors:** Aleš Kopal, Eva Mejzlíková, Jana Lízrová Preiningerová, David Brebera, Olga Ulmanová, Edvard Ehler, Jan Roth

**Affiliations:** ^1^Department of Neurology, Regional Hospital Pardubice, Kyjevska 44, 532 03 Pardubice, Czech Republic; ^2^Department of Ophthalmology, Regional Hospital Pardubice, Kyjevska 44, 532 03 Pardubice, Czech Republic; ^3^Department of Neurology and Center of Clinical Neuroscience, Charles University in Prague, 1st Faculty of Medicine and General University Hospital in Prague, Katerinska 30, 128 21 Prague, Czech Republic; ^4^Department of Mathematics and Quantitative Methods, Faculty of Economics and Administration, University of Pardubice, Studentska 95, 532 10 Pardubice, Czech Republic

## Abstract

Parkinson's disease (PD) is characterized by motor and nonmotor symptoms. Nonmotor symptoms include primarily visual hallucinations (VH). The aim of our study was to establish whether patients with PD and visual hallucinations (PDH+) have structural changes of retina detected by an optical coherence tomography (OCT) in comparison with PD patients without visual hallucinations (PDH−). We examined 52 PD patients (18 with VH, 34 without VH) and 15 age and sex matched healthy controls. Retinal nerve fiber layer (RNFL) thickness and macular thickness and volume were assessed by OCT. Functional impairment of retina was assessed using 2.5% contrast sensitivity test. For OCT outcomes we analyzed 15 PDH+ and 15 PDH− subjects matched for age, gender, and PD duration. For contrast sensitivity we analyzed 8 pairs of patients matched for age, gender, and visual acuity. There was no significant difference in RNFL thickness and macular thickness and macular volume between 15 PDH+ and 15 PDH− subjects, and also between a group of 44 PD patients (both PDH+ and PDH−) and 15 age and gender matched healthy controls. No significant difference was found for 2.5% contrast sensitivity test values between PDH+ and PDH− subjects. Therefore we conclude that functional and structural changes in retina play no role in genesis of VH in PD.

## 1. Introduction

Parkinson's disease (PD) is a slowly progressive neurodegenerative disorder characterized by numerous motor and nonmotor symptoms. Cardinal motor symptoms (bradykinesia, rigidity, rest tremor, and postural instability) develop due to dopaminergic deficiency in striatum. Degree of dopaminergic deficiency correlates with disease duration and progression of motor symptoms [1]. Nonmotor symptoms include vegetative dysfunction, sleep disorders, depression, cognitive dysfunctions, psychosis, and sensory disturbances. Visual hallucinations (VH) are typical feature of psychosis in PD. VH develop in 30–60% of PD patients [2, 3] and their prevalence increases with disease progression [4].

Pathophysiology of hallucinations in PD has not been fully clarified yet. VH may be induced by dopaminergic drugs but may also develop as a natural symptom of PD. In pathogenesis of hallucinations, both peripheral (retinal) and central (association cortex) changes have been discussed [2, 4].

Disturbances of retina, where dopaminergic amacrine cells are located, have been considered [5]. Amacrine cells influence synapses in all retinal layers and are therefore involved in photopic and scotopic vision [6]. Retinal dysfunction may influence image creation and cause its disturbances [5–8]. Morphological changes of multiple cell layers in retina may follow. Changes in retina can be imaged by optical coherence tomography (OCT), which is a noninvasive technique that captures retinal structures in micrometer resolution.

We hypothesized that, as a consequence of functional and structural changes of dopaminergic amacrine cells in retina, there is a significant reduction of RNFL thickness and macular thickness and volume in PD patients with hallucinations (PDH+) compared to PD patients without hallucinations (PDH−). Such result would support the role of retinal involvement in genesis of VH.

## 2. Subjects and Methods

All patients with diagnosis of PD that attended Clinic of Neurology in a Regional Hospital Pardubice from May 2011 to May 2012 were preselected for the study. Only even-numbered subjects from the alphabetically ordered name list of 164 patients were invited to join the study. 71 subjects were included in the study as 11 subjects refused to participate.

The study was approved by a local ethical committee and all subjects gave informed consent prior to the enrollment to the study.

All subjects fulfilled the diagnostic criteria of PD according to the United Kingdom Parkinson's Disease Society Brain Bank [9]. Only subjects with disease onset after 40 years of age were included in the study. Exclusion criteria were history of central nervous system disorder other than PD, inflammatory disorder of the eye in the last 3 months, and history of optic neuritis or vitreoretinal pathology (including glaucoma, diabetic retinopathy, and age-related macular degeneration). Altogether 19 subjects had to be excluded and remaining 52 subjects (18 patients with VH and 34 without VH) were included in the study.

Data on PD history (age, PD onset, and disease duration) were obtained from all the subjects. All patients were assessed by UPDRS motor scale (subscale III) [10] in on-state. Cognitive impairment was tested by Montreal Cognitive Assessment test (MoCA) [11].

PDH+ subjects were defined as patients with repeated occurrence of VH either isolated or in combination with other modality hallucinations. Hallucinations were present at study inclusion or have occurred repeatedly in last 2 years and their severity required continuous antipsychotic treatment or significant modification of antiparkinsonian therapy.

VH and delusions were assessed by MDS-UPDRS (Movement Disorder Society-sponsored revision of the Unified Parkinson's Disease Rating Scale), items Hallucinations and psychosis part I [10]. Rush Hallucination Inventory [12, 13] monitored frequency and type (visual, auditory, tactile, and olfactory) of hallucinations.

PDH− subjects were defined as patients with no history of hallucinations of any type.

Ophthalmological examination included visual acuity (100% contrast), intraocular pressure measurement, and fundus examination for exclusion of vitreoretinal pathology. OCT was performed using Zeiss Stratus OCT Model 3000 after pharmacologic pupillary dilatation. Mean RNFL thickness, mean macular thickness apart from fovea, and total macular volume were assessed for both eyes. Results from the eye with higher signal quality were used for statistical analysis.

Contrast sensitivity test, as a parameter of retinal function, was performed in 15 PDH+ and 30 PDH− patients. Snellen charts with 2.5% contrast at a distance of 3 m were used for testing (Lea Contrast Sensitivity Test) [14, 15]. The charts were illuminated by 6500 K light source from a fixed distance.

## 3. Statistical Analysis

Statsoft Statistica 10 software suite was used for analysis. Pearson correlation coefficient, one-sample* t*-test, independent two-sample* t*-test, and dependent* t*-test for paired samples were used. For OCT data also test-retest variability and test for correlation of repeated examination were performed.

For higher statistical significance, 15 pairs of PDH+ and PDH− matched for age, gender, and disease duration were compared. Control group included 15 healthy subjects in the 6th and 7th decade. Further analysis of results of contrast sensitivity was performed in 8 pairs of patients matched for age, gender, and visual acuity.

## 4. Results

We examined 18 PDH+ patients (10 men, 8 women) and 34 PDH− patients (18 men, 16 women). Average age in PDH+ group was 71.8 years and in PDH− group was 68 years. Average duration of the disease in PDH+ group was 10.2 years and in PDH− group 7.5 years. For more details about subjects see Tables 1 and 2. Pure visual hallucinations were present in 16 patients, 1 patient reported both visual and auditory hallucinations, and 1 patient reported both hallucinations and delusions.

Patients with VH had longer disease duration, higher score in motor scale UPDRS, and lower score in MoCA test compared to patients without hallucinations. Differences in other parameters tested (RNFL thickness, macular thickness, and volume) were not statistically significant (Table 3).

When analyzing the 15 pairs of PDH+ and PDH− matched for age, gender, and disease duration, the UPDRS score was higher (*p* < 0.0001) in PDH+. There was no significant difference in other parameters (RNFL thickness, macular thickness, macular volume, and MoCA score) (Table 4).

There was no significant difference in low contrast vision (2.5% contrast test) between PDH+ and PDH– patients matched for age and gender (test was available for 8 pairs). Mean value of 2.5% contrast sensitivity was 0.39 (SD 0.2) in PDH+ and 0.23 (SD 0.21) in PDH− (*p* = 0.23), CI (−0.13; 0.46).

There was no significant difference in RNFL thickness, macular thickness, and macular volume between PD and normal control group (*p* > 0.05 for all *p*-values) (Table 5).

## 5. Discussion

It has been shown in several studies that patients with PD have lower RNFL thickness, macular thickness, and macular volume than normal controls [16–21] (Table 6). However, other studies with OCT failed to find significant difference between PD patients and healthy subjects [22, 23].

Controversial results may be predetermined by subject selection, small sample sizes, or variable sensitivity of OCT instruments.

The very first OCT study investigating VH in PD patients, Lee et al. [24] (with mean age and PD duration similar to our study), used high-resolution spectral domain OCT for comparison of 56 PD patients in three subgroups: no VH and no dementia (VH−D−), with VH and no dementia (VH+D−), and with VH and with dementia (VH+D+) to 30 healthy controls. The RNFL was thinnest in the group VH+D−, followed by the group VH+D+, and the group VH−D−.

Our results did not show any significant difference in RNFL thickness and macular thickness and volume, neither between PDH+ and PDH− subgroups nor between 15 matched pairs of PDH+ and PDH−. No significant difference was found between patients and healthy controls matched by age and gender.

In our study, mean age in PD subjects was higher than in studies that reported difference between PD and control groups [16, 18–20]. Younger patients with shorter disease duration may have different results than a group of older patients or a group of patients with longer duration of PD or cohorts with uneven proportion of disease stages. In addition, retinal thickness differs between male and female subjects of the same age.

If structural changes of retina, as a consequence of neurodegeneration, are present since the early stages of PD, such changes should persist or progress in time. According to our knowledge, such prospective longitudinal studies are missing.

Reduced contrast sensitivity in PD patients compared to controls has been reported in several studies [14, 15, 25]. In our study, no significant difference at 2.5% contrast sensitivity test was found between PDH+ and PDH− groups. Incidence of hallucinations was not related to values of 2.5% contrast sensitivity test. We conclude that contrast sensitivity, a parameter of retinal function, plays no role in the incidence of hallucinations in PD. This finding correlates with the results of morphological parameters tested by OCT where no significant difference was found between PDH+ and PDH− groups. Structural and functional parameters were in concordance.

Analysis of PDH+ patients revealed significantly longer disease duration, higher score in UPDRS, and lower score in MoCA compared with PDH− group. Incidence of hallucinations thus increases with disease duration. In analysis of pairs of PDH+ and PDH− patients we found significantly higher score in UPDRS in PDH+ subjects compared with PDH−. Patients with hallucinations had more severe motor impairment than PD subjects of same age, gender, and disease duration without hallucinations. Risk of hallucinations is therefore greater in patients with more severe motor impairment. Cognitive decline showed no influence on presence of hallucinations.

Our data show that patients with and without VH cannot be distinguished by means of OCT. No morphological or functional changes of retina were found in PDH+ in comparison with PDH−. This can be explained by several factors. We can presume that occurrence of VH is underlined by morphological or functional changes at cerebral level and does not relate to retina. Or we can speculate that VH are related to complex dysfunction on retinal level but is not represented by total retinal thinning chosen as an outcome in this study.

The strength of the study is a rigorous matching by age, gender, and disease duration, which creates homogeneous cohorts.

Our conclusion is further supported by no difference in low contrast sensitivity testing between PDH+ and PDH− subgroups. According to our results, we also infer that presence of VH may not be primarily related to cognitive decline.

## 6. Conclusion

Besides recently published study of Lee at al. [24], this is the only study dealing with structural and functional parameters of retina in PD patients with and without VH. Neither structural parameters of retina tested by means of OCT nor contrast sensitivity as a functional measure differ between PD patients with VH and without VH.

We conclude that functional and structural changes in retina are not related to VH in PD.

Further studies should focus on possible changes in segmented layers of retina, specifically in a layer with amacrine cells.

## Figures and Tables

**Table 1:** Demographic data.

	PDH+		PDH−	
	Men	Women	Men	Women
	*N* = 10	*N* = 8	*N* = 18	*N* = 16
Mean age (SD)	69 (9.6)	74.6 (5.7)	67.6 (8.9)	68.3 (7.9)
Range (years)	52–83	65–81	43–80	49–79

Mean disease duration (SD)	10.2 (4.2)	10.1 (5.1)	6.7 (3.5)	8.2 (4.4)
Range (years)	4–18	3–19	2–13	3–16

PDH+: group of Parkinson's disease with hallucinations; PDH−: group of Parkinson's disease without hallucinations; SD: standard deviation.

**Table 2:** Demographic data.

	Paired PDH+		Paired PDH−	
	Men	Women	Men	Women
	*N* = 9	*N* = 6	*N* = 9	*N* = 6
Mean age (SD)	69.9 (9.7)	72.5 (4.9)	68.9 (6.1)	71.3 (4.6)
Range (years)	52–83	65–79	61–78	65–78

Mean disease duration (SD)	9.3 (3.3)	9.5 (5.7)	8 (3.1)	8.2 (4.7)
Range (years)	4–14	3–19	4–13	3–15

Paired PDH+: paired group of Parkinson's disease with hallucinations; paired PDH−: paired group of Parkinson's disease without hallucinations; SD: standard deviation.

**Table 3:** Two-sample *t*-test for population mean in groups PDH+ and PDH−.

	PDH+	PDH−	*t*-test	*F*-test
	*n* = 18	*n* = 34	*p*-value	*p*-value
Age (years)	71.5 (SD 8.39)	67.76 (SD 8.24)	0.13	0.89
PD duration (years)	10.17 (SD 4.45)	7.12 (SD 4.02)	**0.02**	0.6
RNFL (*μ*m)	97.9 (SD 10.86)	97.85 (SD 10.64)	0.99	0.89
Macula T (*μ*m)	249.96 (SD 18.21)	252.84 (SD 12.66)	0.51	0.07
Macula V (mm^3^)	6.85 (SD 0.45)	6.89 (SD 0.34)	0.67	0.18
UPDRS	28.06 (SD 9.85)	16.65 (SD 6.61)	**p** < 0.001	0.05
MoCA	17.39 (SD 6.58)	21.35 (SD 3.27)	**0.01**	*p* < 0.001

PDH+: Parkinson's disease with hallucinations; PDH−: Parkinson's disease without hallucinations; *n*: number of patients; SD: standard deviation; RNFL: retinal nerve fiber layer; macula T: macula thickness without fovea; macula V: macula volume; MoCA: Montreal Cognitive Assessment; UPDRS: Unified Parkinson's Disease Rating Scale.

**Table 4:** Paired two-sample *t*-test for population mean in groups PDH+ and PDH−.

	*n* = 15	*t*-test
		*p*-value
PDH+ age (years)	70.93 (SD 8.02)	0.30
PDH− age (years)	69.87 (SD 5.5)	

PDH+ disease duration (years)	9.4 (SD 4.24)	0.08
PDH− disease duration (years)	8.07 (SD 3.67)	

PDH+ RNFL (*μ*m)	96.79 (SD 11.11)	0.55
PDH− RNFL (*μ*m)	93.94 (SD 12.94)	

PDH+ macula T (*μ*m)	248.29 (SD 14.35)	0.09
PDH− macula T (*μ*m)	256.35 (SD 12.02)	

PDH+ macula V (mm^3^)	6.81 (SD 0.32)	0.14
PDH− macula V (mm^3^)	6.99 (SD 0.32)	

PDH+ UPDRS	27.33 (SD 10.03)	**p** < 0.0001
PDH− UPDRS	14.4 (SD 5.37)	

PDH+ MoCA	18.4 (SD 6.65)	0.19
PDH− MoCA	21.47 (SD 3.31)	

PDH+: Parkinson's disease with hallucinations; PDH−: Parkinson's disease without hallucinations; *n*: number of paired patients; SD: standard deviation; RNFL: retinal nerve fiber layer; macula T: macula thickness without fovea; macula V: macula volume; MoCA: Montreal Cognitive Assessment; UPDRS: Unified Parkinson's Disease Rating Scale.

**(a):** 

60–69 years	PD	Healthy controls	*t*-test	*F*-test
	*n* = 26	*n* = 8	*p*-value	*p*-value
RNFL (*μ*m)	102.4	97.22	0.20	0.19
	(SD 10.55)	(SD 6.54)		

Macula T (*μ*m)	256.71	252.56	0.52	0.71
	(SD 16.31)	(SD 13.98)		

Macula V (mm^3^)	6.98	6.86	0.47	0.70
	(SD 0.43)	(SD 0.37)		

**(b):** 

70–79 years	PD	Healthy controls	*t*-test	*F*-test
	*n* = 18	*n* = 7	*p*-value	*p*-value
RNFL (*μ*m)	91.33	93.35	0.54	0.16
	(SD 6.34)	(SD 9.71)		

Macula T (*μ*m)	247.48	249.84	0.68	0.49
	(SD 11.86)	(SD 4.41)		

PD: Parkinson's disease; SD: standard deviation; *n*: number of patients; RNFL: retinal nerve fiber layer; macula T: macula thickness without fovea; macula V: macula volume.

**(c):** 

60–79 years	PD	Healthy controls	*t*-test	*F*-test
	*n* = 44	*n* = 15	*p*-value	*p*-value
RNFL (*μ*m)	97.87	95.42	0.41	0.29
	(SD 10.53)	(SD 8.11)		

Macula T (*μ*m)	252.93	252.29	0.71	0.70
	(SD 15.21)	(SD 13.74)		

Macula V (mm^3^)	6.91	6.82	0.43	0.83
	(SD 0.38)	(SD 0.36)		

Note: as the age and observed RNFL and macula parameters exhibit the same correlation in all groups, we decided to join the age groups (initially observed by decades) together and perform the statistics on larger samples. For all the tested parameters the *p*-values are significantly more than critical level of 0.05; that is, no statistically significant difference was found in the samples.

**Table 6:** 

Author	Number of patients with PN	Number of controls	Results	*p*-value	OCT device
Inzelberg et al. 2004 [18]	10	10	Thinning of peripapillar RNFL thickness in the inferotemporal area	0.0003	Not mentioned

Altintaş et al. 2008 [16]	17	11	Thinning of RNFL thickness and reduce of macula volume	<0.05	OCT model 3000 software version A1.1

Hajee et al. 2009 [17]	23	17	Thinning of paramacular inner retinal layer	0.01	Fourier-domain OCT

Moschos et al. 2011 [20]	16	20	Thinning of RNFL thickness in the inferior and temporal area	<0.0001 and 0.0045	OCT Stratus Model 3000

Kirbas et al. 2013 [19]	42	40	Thinning of RNFL thickness in the temporal quadrant	0.001	Cirrus HD SD-OCT

Morgia et al. 2013 [21]	41	86	Thinning of RNFL layer in the temporal quadrant	0.004	Stratus OCT software version 4.0.1

OCT: optical coherence tomography; PN: Parkinson's disease; RNFL: retinal nerve fiber layer.
